# Which Children with Autism Spectrum Disorder Have Abnormal Brain MRI Findings? Clinical Correlates in a 1884-Patient Cohort

**DOI:** 10.3390/neurolint18090159

**Published:** 2026-08-22

**Authors:** Viswabhaskar Susarla, Mariah George, Ananthi Rathinam, Danish Bhatti

**Affiliations:** 1 Kids Neuro Care LLC, 10931 Dylan Loren Cir, Orlando, FL 32825, USA; 2 Deptartment of Neurology, University of Central Florida College of Medicine, 6850 Lake Nona Blvd., Orlando, FL 32827, USA; ma030549@ucf.edu (M.G.);

**Keywords:** autism spectrum disorder, brain magnetic resonance imaging, clinical decision-making, incidental findings, selection bias, retrospective cohort, diagnostic testing

## Abstract

**Background:** Brain MRI in patients with autism spectrum disorder (ASD) is generally considered when additional neurologic, developmental, or syndromic concerns warrant structural evaluation. In the study practice, ASD alone was not used as the reason to obtain MRI. We examined documented MRI use and broad result coding while explicitly separating selection for imaging from abnormal-result status. **Methods:** This retrospective specialty-practice cohort included 1884 patients with ASD. The primary outcome was the proportion of documented MRI examinations coded abnormal. Secondary analyses examined associations of age, sex, seizure history, EEG status, and genetic test status with abnormal versus normal MRI coding and with documented MRI utilization. MRI categories and the pediatric restriction were exploratory and sensitivity analyses. **Results:** An MRI result was recorded for 704 patients (37.4%; 95% CI 35.2–39.6); 322 were coded abnormal (45.7%; 95% CI 42.0–49.5). The abnormal-result models had low in-sample discrimination (AUC 0.528 and 0.553), and adjusted confidence intervals for all measured variables included 1.00; these results do not demonstrate equivalence between clinical groups. Seizure history was associated with documented imaging in the full cohort (aOR 2.47, 95% CI 1.94–3.14), and abnormal EEG was associated in the exploratory complete-case utilization model (aOR 2.83, 95% CI 1.93–4.14). **Conclusions:** Broad MRI abnormalities were frequent among patients selected for imaging because of additional clinical concerns, but a broad abnormal code is not equivalent to clinically actionable yield. The routinely captured variables available in this dataset were insufficient to distinguish abnormal from normal MRI coding. These findings favor individualized MRI decisions based on the clinical question prompting imaging and motivate future studies with richer neurologic and developmental phenotyping.

## 1. Introduction

Autism spectrum disorder (ASD) is a heterogeneous neurodevelopmental condition diagnosed clinically. Medical evaluation seeks co-occurring neurological disorders and potentially explanatory genetic, metabolic, or structural conditions; structural MRI is not used to establish the behavioral diagnosis. Contemporary guidance emphasizes targeted medical evaluation rather than routine neuroimaging [1,2,3,4]. The American Academy of Pediatrics identifies atypical regression, microcephaly or macrocephaly, seizures, intracranial manifestations of genetic disorders, an abnormal neurological examination, and other specific clinical indications as situations in which MRI may be considered [1]. For patients with co-occurring global developmental delay or intellectual disability, recent guidance similarly ties MRI to neurologic features rather than to the neurodevelopmental diagnosis alone [5].

Reported MRI abnormality rates in ASD vary substantially because referral thresholds, patient populations, imaging protocols, and outcome definitions differ. When the endpoint is definite or clinically significant pathology, reported yields are lower: Byrne et al. found significant abnormalities in 7.2% of 181 children, and Cooper et al. found the lowest prevalence of definite pathology when ASD alone was the indication [6,7]. Other clinical series further illustrate the effect of population and protocol differences [8,9]. Studies counting minor, nonspecific, or incidental findings have reported any-abnormality proportions near 48–55% [10,11]. In nonclinical pediatric cohorts, incidental findings are common but only a smaller subset prompt referral, underscoring that any-abnormality is not synonymous with clinical actionability [12,13].

Research neuroimaging has identified group-level structural and functional differences across the lifespan, but those observations do not establish MRI as an individual diagnostic or screening test [14]. Clinical studies must therefore distinguish three questions: why MRI is obtained, what the report records, and whether a finding changes diagnosis or management. A characteristic may influence selection for imaging without increasing the probability of a clinically meaningful result among selected patients.

Reported MRI yields in ASD are difficult to compare because two distinct processes are often blended: which patients are selected for imaging and what is counted as an abnormal MRI result. The present study was designed to separate those processes. The primary question was what proportion of patients with a documented MRI examination had a result coded abnormal. The two secondary questions were (1) whether routinely documented demographic and neurodiagnostic variables were associated with abnormal versus normal MRI coding and (2) whether those variables were associated with documented MRI utilization in the full cohort. This distinction is intended to clarify why a characteristic can influence the decision to image without necessarily discriminating abnormal from normal findings after imaging has already been selected. MRI subtype categories and a pediatric-only analysis were treated as exploratory description and sensitivity analysis.

## 2. Materials and Methods

### 2.1. Study Design and Setting

This retrospective cohort study used a de-identified clinical dataset derived from patients evaluated at Kids Neuro Care LLC, a specialized pediatric neurology practice. Data were obtained from retrospective review of electronic medical records covering the institutional study period. The report follows the Strengthening the Reporting of Observational Studies in Epidemiology (STROBE) recommendations [15].

Practice context is important to interpretation of the cohort. An ASD diagnosis by itself was not used as the reason to obtain brain MRI at this specialty practice; imaging was obtained when the treating physician identified additional clinical concerns warranting structural evaluation. The de-identified analytic dataset did not retain the specific patient-level indication for each MRI, so those individual referral reasons could not be reconstructed or modeled.

### 2.2. Ethics

On 28 July 2026, the University of Central Florida Institutional Review Board determined the study to be exempt human-subjects research under Category 4(ii) (IRB ID: STUDY00009410) and approved a waiver of HIPAA authorization. The approved protocol was titled “Which Children With Autism Spectrum Disorder Have Abnormal Brain MRI Findings? A 1884-Patient Cohort Study”; the manuscript title was subsequently revised for precision without changing the protocol, cohort, outcomes, or analyses.

### 2.3. Participants

The analytic cohort contained 1884 unique records with a documented clinical diagnosis of ASD and valid age and sex data. The de-identified dataset did not contain sufficient information to verify the use of a specific standardized diagnostic instrument for every patient. Duplicate records and records without adequate demographic or neurodiagnostic information were excluded during construction of the master dataset. The age range was 1.75–28.00 years; 170 patients (9.0%) were aged 18 years or older. The full cohort was retained because the source pediatric neurology practice continued to evaluate some young adults. A sensitivity analysis restricted to patients younger than 18 years was used to assess whether inclusion of young adults materially altered the abnormal-MRI conclusions.

### 2.4. Variables and Outcome Definitions

Age was analyzed continuously in years and descriptively in the categories 0–4, 5–9, 10–14, 15–17, and 18 years or older. Sex was recorded as either male or female. Seizure history was recorded as present or absent based on documentation in the clinical dataset; detailed seizure classification and temporal relationship to MRI were not uniformly available.

MRI status was coded as not tested, normal, or abnormal. Documented MRI utilization was operationally defined by the presence of a normal or abnormal brain MRI result in the dataset; records coded not tested were classified as having no documented MRI result. The primary outcome was the proportion of documented MRI examinations coded abnormal. Because complete report-level adjudication and downstream management data were unavailable, the broad source-level abnormal code was analyzed as a radiologic result category rather than as a measure of etiologic, clinically significant, actionable, or management-changing yield.

EEG and genetic/chromosomal testing were each coded as not tested, normal, or abnormal. For binary analyses, patients without a completed test were treated as missing rather than as normal. A genetic abnormality reflected the source dataset code and did not establish that every finding was pathogenic, causal, or explanatory for ASD; contemporary variant-interpretation standards distinguish pathogenic and likely pathogenic variants from variants of uncertain significance and benign categories [16].

Structured MRI category and finding-class variables were available for a subset of abnormal results. Categories included white matter changes or gliosis, cyst or cerebrospinal fluid-space findings, ventriculomegaly or volume loss, vascular or hemorrhagic findings, Chiari malformation or tonsillar ectopia, cortical malformation, corpus callosum anomaly, mesial temporal or hippocampal abnormality, pituitary/optic/sellar findings, posterior fossa findings, and other structural or nonspecific findings. These were source-level category labels; no central image review, report-level reclassification, or retrospective assignment of clinical significance was performed.

### 2.5. Outcomes and Analysis Hierarchy

The primary outcome was the proportion of abnormal-result codes among patients with a documented MRI result. Secondary outcome one was abnormal versus normal MRI coding among those patients. Its core association model included age, sex, and seizure history; an extended complete-case model added EEG and genetic test status. Secondary outcome two was documented MRI utilization in the full cohort, defined as the presence versus absence of a recorded normal or abnormal MRI result. Its core model included age, sex, and seizure history; an exploratory extended complete-case model added EEG and genetic test status. Structured MRI categories were summarized only within the subset with available subtype labels. To assess whether availability of subtype labels was associated with measured patient characteristics, the 159 abnormal MRI records with structured subtype coding were compared with the 163 abnormal MRI records without subtype coding. The abnormal-result analyses were also repeated in patients younger than 18 years; both the pediatric core model and the pediatric extended complete-case model were specified to mirror the main analysis.

### 2.6. Statistical Analysis

Continuous variables are summarized as mean and standard deviation. The abnormal-result and recorded MRI utilization proportions are reported with exact 95% binomial confidence intervals. Age was compared with Welch’s independent-samples *t* test. Categorical variables were compared using Pearson’s chi-square test; Fisher’s exact test was examined for 2 × 2 tables. Effect sizes were summarized using odds ratios and Cramer’s V where appropriate.

Separate binomial logistic regressions estimated adjusted odds ratios (aORs) and 95% confidence intervals for the two secondary outcomes. Normal MRI coding was the reference in abnormal-result models, and no documented MRI result was the reference in utilization models. Male sex, no seizure history, normal EEG, and normal genetic testing were the predictor reference categories. Complete-case analysis was used when EEG and genetic variables were included; untested patients were not recoded as normal. Model discrimination was summarized with the in-sample area under the receiver operating characteristic curve (AUC), and variance inflation factors were examined for multicollinearity. For the exploratory subtype-coding missingness analysis, age was compared between coded and uncoded abnormal MRI records using Welch’s *t* test, and categorical characteristics were compared using Pearson’s chi-square test or Fisher’s exact test as appropriate, with effect sizes reported where applicable. Statistical significance was defined as a two-sided *p* value < 0.05. Secondary, exploratory, and sensitivity analyses were interpreted cautiously without multiplicity adjustment. Confidence intervals that included 1.00 were interpreted as a lack of statistically supported association, not as evidence of equivalence or proof of no clinically relevant effect. Because abnormal MRI coding was common, adjusted odds ratios should not be interpreted as prevalence ratios or risk ratios. The models were descriptive association models and were not developed or validated as clinical prediction rules. Analyses were performed using jamovi version 2.7 (the jamovi project, Sydney, Australia).

## 3. Results

### 3.1. Cohort Characteristics and MRI Utilization

The cohort included 1884 patients with a mean age of 10.64 years (SD 4.81; range 1.75–28.00). There were 1424 males (75.6%) and 460 females (24.4%); 339 patients (18.0%) had a documented seizure history. EEG was recorded as completed in 1561 patients (82.9%), and 285 of those tested (18.3%) had an abnormal result. Genetic/chromosomal testing was recorded as completed in 1011 patients (53.7%), and 371 of those tested (36.7%) had an abnormal result.

A normal or abnormal brain MRI result was recorded for 704 patients (37.4%; 95% CI 35.2–39.6); 1180 records had no MRI result recorded. Patients with a documented MRI result were slightly older than those without one (10.97 vs. 10.45 years; *p* = 0.021) and more frequently had a seizure history (26.8% vs. 12.7%; *p* < 0.001). EEG and genetic testing were also more frequently recorded as completed among patients with documented MRI results (Table 1). In the practice setting, MRI was obtained for clinical concerns beyond the ASD diagnosis itself; these comparisons therefore describe patterns of neurologic evaluation and imaging utilization rather than autism-driven screening.

### 3.2. Primary Outcome: Abnormal MRI Proportion

Among 704 patients with a documented MRI result, 322 had an abnormal code and 382 had a normal code. The primary outcome proportion was 45.7% (95% CI 42.0–49.5). Thus, broad radiologic abnormalities were common among patients selected for MRI in this specialty-practice cohort. The estimate represents any abnormal source-level code and should not be interpreted as the proportion with an etiologic or management-changing finding.

In unadjusted comparisons, mean age was similar in the abnormal- and normal-code groups (11.09 vs. 10.87 years; Welch t = 0.63, *p* = 0.531; Cohen’s d = 0.05). The abnormal code proportions were 49.1% in females and 44.7% in males (*p* = 0.318), 46.6% in patients with a seizure history and 45.4% in those without (*p* = 0.791), 45.5% among patients with abnormal EEG and 43.9% among those with normal EEG (*p* = 0.718), and 47.6% among patients with an abnormal genetic test code and 39.4% among those with a normal genetic test (*p* = 0.083). The overall age-group comparison was not statistically significant (*p* = 0.086) (Table 2).

**Table 2:** Characteristics according to broad abnormal versus normal MRI coding.

Characteristic	Abnormal MRI (n = 322)	Normal MRI (n = 382)	*p* Value
Age, mean (SD), years	11.09 (4.93)	10.87 (4.28)	0.531
Female sex, n (%)	82 (25.5)	85 (22.3)	0.318
Seizure history, n (%)	88 (27.3)	101 (26.4)	0.791
Abnormal EEG, n/N tested (%)	85/279 (30.5)	102/350 (29.1)	0.718
Abnormal genetic test, n/N tested (%)	88/198 (44.4)	97/266 (36.5)	0.083
EEG not tested, n (%)	43 (13.4)	32 (8.4)	—
Genetic test not performed, n (%)	124 (38.5)	116 (30.4)	—

Binary EEG and genetic comparisons are restricted to tested patients; untested patients were not treated as normal. An abnormal MRI code was not adjudicated for clinical significance. MRI, magnetic resonance imaging; EEG, electroencephalography.

### 3.3. Secondary Outcome 1: Factors Associated with Abnormal MRI Coding

The core model included all 704 patients with a documented MRI result and was not statistically significant overall (likelihood-ratio chi-square = 1.44, 3 df, *p* = 0.696; AUC = 0.528). Estimates for age, sex, and seizure history were close to 1.00, with confidence intervals that included 1.00 (Table 3). The extended complete-case model included 422 patients with available MRI, EEG, and genetic test results and was also not statistically significant overall (likelihood-ratio chi-square = 4.41, 5 df, *p* = 0.492; AUC = 0.553). Estimates for abnormal EEG and an abnormal genetic test code were imprecise and also included 1.00. Variance inflation factors were 1.01–1.12. The AUC values indicate that the routinely captured variables available in these models provided little discrimination between broad abnormal and normal MRI coding. They do not establish clinical equivalence between groups or exclude associations with unmeasured neurologic features.

### 3.4. Secondary Outcome 2: Recorded MRI Utilization

In the core utilization model using the full cohort, seizure history was associated with greater odds of having a recorded MRI result (aOR 2.47, 95% CI 1.94–3.14), whereas age and sex were not. The model was statistically significant overall (likelihood-ratio chi-square = 59.6, 3 df, *p* < 0.001) but had modest in-sample discrimination (AUC = 0.589).

The exploratory extended complete-case utilization model included 901 patients with available EEG and genetic test results. Seizure history (aOR 2.10, 95% CI 1.44–3.08) and abnormal EEG (aOR 2.83, 95% CI 1.93–4.14) were associated with a documented MRI result. The estimates for genetic test status, age, and sex included 1.00. Model discrimination remained modest (AUC = 0.656). These associations are consistent with greater MRI utilization among patients undergoing more intensive neurologic evaluation; because the specific patient-level indication and test chronology were unavailable, they describe care-pathway associations rather than causal referral effects (Table 4).

**Table 4:** Logistic regression models for documented MRI utilization.

Model	Predictor	Adjusted OR	95% CI	*p* Value
Core utilization model (N = 1884)	Age, per year	1.01	0.99–1.03	0.201
	Female vs. male	0.99	0.80–1.24	0.955
	Seizure history, yes vs. no	2.47	1.94–3.14	<0.001
Exploratory extended complete-case model (n = 901)	Age, per year	1.01	0.98–1.04	0.455
	Female vs. male	0.78	0.57–1.07	0.130
	Seizure history, yes vs. no	2.10	1.44–3.08	<0.001
	Abnormal vs. normal EEG	2.83	1.93–4.14	<0.001
	Abnormal vs. normal genetic test	1.22	0.91–1.62	0.180

No documented MRI result was the reference outcome. Presence of a documented MRI result was defined by a normal or abnormal code. Associations describe observed care and documentation patterns and do not establish patient-level indication, chronology, or causation. OR, odds ratio; CI, confidence interval; MRI, magnetic resonance imaging; EEG, electroencephalography.

### 3.5. Exploratory Description of MRI Categories in the Coded Subset

Structured MRI category data were available for 159 of 322 abnormal results (49.4%); 163 abnormal results (50.6%) lacked subtype coding. Within the coded subset, white matter changes or gliosis were the most frequent category (49/159, 30.8%), followed by cyst or cerebrospinal fluid-space findings (30/159, 18.9%) and ventriculomegaly or volume loss (15/159, 9.4%) (Table 5). Source-level finding-class coding categorized 83 results (52.2%) as acquired/other, 32 (20.1%) as congenital/developmental, 30 (18.9%) as incidental/benign, 10 (6.3%) as nonspecific/other, and 4 (2.5%) as other. The subtype percentages therefore describe documentation within the coded subset rather than prevalence across all abnormal MRI examinations. Because subtype coding was absent for approximately half of abnormal results, informative missingness remains possible even if measured characteristics appear similar between coded and uncoded records.

Percentages use the 159 abnormal results with structured category coding as the denominator. Because 163 of 322 abnormal results lacked subtype coding, the category distribution is descriptive of the coded subset and should not be generalized to all abnormal MRI examinations. CSF, cerebrospinal fluid; MRI, magnetic resonance imaging.

In an exploratory comparison of abnormal MRI records with versus without structured subtype coding, the 163 uncoded records involved older patients than the 159 coded records (mean age 11.8 vs. 10.4 years; Welch t (318) = 2.49, *p* = 0.013; Cohen’s d = 0.277). Sex distribution did not differ significantly between groups (chi-square (1) = 1.32, *p* = 0.251; Cramer’s V = 0.064). By contrast, seizure history was more frequent in the uncoded group than in the coded group (35.6% vs. 18.9%; chi-square (1) = 11.3, *p* < 0.001; Cramer’s V = 0.188), and the distributions of EEG status (chi-square (2) = 17.2, *p* < 0.001; Cramer’s V = 0.231) and genetic-testing status (chi-square (2) = 9.19, *p* = 0.010; Cramer’s V = 0.169) also differed. These measured differences indicate that availability of structured subtype coding was not exchangeable across the abnormal-MRI subgroup and reinforce that Table 5 describes the coded subset only.

### 3.6. Sensitivity Analysis Restricted to Patients Younger than 18 Years

Among 639 patients younger than 18 years with a documented MRI result, 288 had an abnormal code (45.1%; 95% CI 41.2–49.0), compared with 45.7% in the full imaged cohort. Of the 704 patients with documented MRI results, 65 were aged 18 years or older; 34 of those 65 (52.3%) had an abnormal code. Excluding young adults therefore changed the overall abnormal proportion by only 0.6 percentage points. The pediatric extended complete-case model included 393 patients and was not statistically significant overall (likelihood-ratio chi-square = 5.06, 5 df, *p* = 0.409; AUC = 0.561), closely paralleling the full extended model (AUC = 0.553). All confidence intervals included 1.00: age (aOR 0.98, 95% CI 0.93–1.05), female sex (aOR 1.46, 95% CI 0.91–2.34), seizure history (aOR 0.95, 95% CI 0.58–1.54), abnormal EEG (aOR 1.19, 95% CI 0.75–1.91), and an abnormal genetic test code (aOR 1.28, 95% CI 0.84–1.95). These results indicate that inclusion of young adults did not materially change the abnormal-MRI proportion or the interpretation of the extended model.

The pediatric core abnormal-result model included all 639 patients younger than 18 years with a documented MRI result and was not statistically significant overall (likelihood-ratio chi-square = 1.19, 3 df, *p* = 0.755; AUC = 0.516). The estimates were age (aOR 0.992, 95% CI 0.949–1.04; *p* = 0.709), female sex (aOR 1.21, 95% CI 0.839–1.74; *p* = 0.307), and seizure history (aOR 1.002, 95% CI 0.698–1.44; *p* = 0.991). The pediatric core model therefore closely paralleled the full-cohort core model (AUC 0.516 vs. 0.528), with no measured predictor showing a statistically supported independent association with broad abnormal MRI coding.

## 4. Discussion

### 4.1. Principal Findings

In this large specialty-practice ASD cohort, a normal or abnormal MRI result was documented for just over one-third of patients, and 45.7% of those results carried an abnormal source-level code. Because ASD itself was not used as the reason to obtain MRI in the study practice, this proportion is best understood as the frequency of broad MRI abnormalities among patients selected for imaging because of additional clinical concerns. That distinction is clinically meaningful: abnormalities were common once MRI was considered warranted, yet the routinely captured demographic and neurodiagnostic variables available in the dataset did not reliably distinguish which imaged patients would have an abnormal rather than normal code. By contrast, seizure history and, in the exploratory complete-case model, abnormal EEG were associated with documented MRI utilization. The findings therefore separate two related but different questions—who enters the imaging pathway and what the MRI subsequently shows.

The 45.7% abnormal code proportion is higher than yields reported in studies that restricted outcomes to definite or clinically significant pathology. Byrne et al. reported significant abnormalities in 7.2% of imaged children, and Cooper et al. found the lowest definite pathology prevalence when ASD alone was the imaging indication [6,7]. Conversely, studies applying broader abnormality definitions reported rates of 48% and 55%, closer to the present estimate [10,11]. Direct comparison is not valid because the present study used a source-level any abnormal code, did not centrally re-read images, and could not adjudicate clinical significance or management consequences.

The study should therefore be read as a description of MRI findings within a clinically selected neurologic population rather than as an estimate of the yield of MRI performed because of ASD. The 45.7% broad abnormality proportion is itself a notable real-world observation, particularly because prior reports using broader definitions of abnormality have produced proportions in a similar range. At the same time, the present data cannot determine which individual abnormalities explained symptoms, altered treatment, or were incidental; that question requires report-level and management-level adjudication.

### 4.2. Selection for MRI Versus Abnormal MRI Status

The utilization analyses show that care and documentation patterns can be strong even when association with a broad result code is weak. Epilepsy is a recognized co-occurring condition in ASD [17], making the association between seizure history and a recorded MRI result clinically plausible. In the exploratory complete-case model, abnormal EEG was also associated with a recorded MRI result. However, the dataset did not record the indication for MRI or the temporal order of testing. These findings therefore describe associations within a care pathway and cannot be converted into causal referral rules.

The absence of the specific MRI indication is the most important unmeasured feature for interpreting the abnormal-result models. In routine practice, the decision to image incorporates information that is far richer than the binary variables available here. Neurological examination findings, focal symptoms, developmental regression, head-growth trajectory, seizure semiology, intellectual or global developmental disability, and syndromic features may contribute to the clinical decision before the patient ever enters the imaged subgroup. Seizure history and EEG status capture only part of that process. Conditioning the abnormal-result analysis on patients who were already selected for MRI can therefore attenuate or obscure associations that would be apparent in a more completely phenotyped cohort.

The abnormal-result models had in-sample AUC values close to 0.5, indicating little discrimination by the available binary variables within this selected sample. This does not establish that the groups are equivalent, rule out modest associations, or show that seizure features are unimportant in an individual patient. Conditioning the analysis on having a recorded MRI result may also introduce selection bias. The dataset lacked neurological examination findings, seizure semiology and focality, developmental regression, head circumference, intellectual disability, tone abnormalities, detailed genotype, and the clinical question prompting MRI. Studies have shown that an abnormal neurological examination or a known genetic or metabolic condition can help identify groups with a higher incidence of significant findings [6,7].

The AUC values of 0.528 and 0.553 should be interpreted as evidence that the measured variables, in the combinations available in this dataset, are poor discriminators of broad abnormal versus normal coding. They are not evidence that patients with and without seizures, EEG abnormalities, genetic findings, or other neurological features are clinically equivalent. No equivalence or non-inferiority analysis was performed. Future models incorporating the actual imaging indication and richer neurological and developmental phenotypes may have substantially different discriminatory performance.

### 4.3. Interpretation of MRI Categories

White matter changes or gliosis and cyst or cerebrospinal fluid-space findings were the most frequent source-level categories among results with subtype coding. Each label can encompass findings with very different implications. Incidental findings also occur in nonclinical pediatric MRI cohorts [12,13], so an imaging abnormality should not automatically be presented as an explanation for ASD or as a reason to change management. In this study, subtype data were missing for 163 of 322 abnormal results, and the available labels were not adjudicated. The category distribution therefore cannot be generalized to all abnormal results or used to estimate actionable yield.

The missing subtype labels materially constrain interpretation of Table 5. Structured category data were present for only 49.4% of abnormal MRI records, and the coded and uncoded groups differed on several measured characteristics: the uncoded group was older and had a higher prevalence of seizure history, while EEG-status and genetic-testing-status distributions also differed; sex distribution was similar. This pattern argues against treating the 159 coded records as a representative sample of all 322 abnormal MRI examinations. The percentages in Table 5 should therefore be interpreted as the distribution of documented categories within the coded subset, not as prevalence estimates for the full abnormal-MRI group. These comparisons characterize measured differences in documentation availability but cannot identify the underlying reason that subtype coding was absent.

### 4.4. Clinical Implications

For clinical practice, this study should not be converted into an MRI decision rule. The data do not support routine MRI solely because ASD is present, but the null association models also should not be used to withhold MRI when a recognized neurologic or syndromic indication exists. The relevant bedside question remains whether the patient’s history, examination, seizure phenotype, regression pattern, head growth, or genetic syndrome creates a specific diagnostic question whose answer could alter counseling, surveillance, treatment, or referral [1,2,3,4,5,6,7].

The wording around routine MRI is intended to contextualize the findings within existing clinical guidance, not to create a decision rule from the present models. In this practice, ASD alone did not trigger MRI; imaging followed an additional clinical concern. The present results are therefore more useful for understanding MRI findings within an indication-selected specialty population than for arguing either for or against MRI as a routine ASD test. A null coefficient should not override a specific neurologic or syndromic indication, and the 45.7% broad abnormality proportion should not itself be used as a screening threshold.

When MRI is considered, potential benefit should be weighed against feasibility and downstream burden. Some children require sedation or anesthesia to complete MRI, which entails dedicated assessment, monitoring, and recovery standards [18]. Broad or incidental findings may also trigger follow-up imaging or consultation [12,13]. This cohort did not measure sedation, follow-up, family burden, or management change, so it cannot quantify those benefits or harms.

Future studies can build directly on these findings by prospectively recording the clinical question that prompted MRI and adjudicating every examination for clinical significance. Richer phenotyping should include neurological examination findings, developmental regression, head circumference trajectory, intellectual or global developmental disability, focal deficits, seizure semiology and focality, detailed genotype, sedation requirement, and downstream changes in diagnosis, treatment, surveillance, counseling, or referral. Such a design would allow investigators to move beyond broad abnormal coding and determine which combinations of clinical features are associated with explanatory or management-changing MRI findings.

### 4.5. Strengths and Limitations

Strengths include the large real-world cohort, the specialty pediatric neurology setting, an explicit distinction between documented MRI utilization and abnormal-result coding, retention of untested EEG and genetic categories as missing rather than normal, a predefined hierarchy of primary and secondary outcomes, multivariable association models, and a pediatric-only sensitivity analysis. The study also demonstrates why selection for imaging and the result of imaging should be analyzed separately and provides a large descriptive estimate of broad MRI abnormalities in patients selected for imaging in routine specialty care.

The most consequential limitation is that the specific patient-level indication for MRI was not retained in the de-identified analytic dataset. This does not mean that MRI lacked a clinical indication: at the study practice, ASD alone was not used as the reason for imaging, and MRI was obtained for additional clinical concerns. However, without the exact indication, the analysis cannot identify which examination finding, regression pattern, head-growth concern, seizure feature, syndrome, or other symptom drove each imaging decision or adjust directly for that selection process. Neurological examination findings, intellectual disability, developmental regression, head circumference, focal deficits, detailed seizure phenotype, and detailed genotype were also unavailable. These omissions are particularly important for the abnormal-result models because those analyses are conditioned on a subgroup that had already been clinically selected for MRI.

Other limitations include unavailable imaging protocols, sedation information, exact test chronology, complete report text, central neuroradiology review, subsequent management, and reliable identification of repeat examinations. The primary outcome therefore combines incidental, nonspecific, and potentially important findings. Structured subtype data were available for only 159 of 322 abnormal results. The coded-versus-uncoded comparison showed differences in age, seizure history, EEG status, and genetic-testing status, supporting caution against generalizing the Table 5 distribution to all abnormal MRI examinations. The presence of an MRI result in the dataset was used as a proxy for utilization and may not capture all imaging performed outside the source practice. External validity should also be considered. A specialized pediatric neurology practice may contain a greater concentration of neurologically complex patients than community pediatrics, developmental-behavioral clinics, multidisciplinary autism centers, or population-based cohorts, so both MRI utilization and the spectrum of abnormalities may differ across settings. The pediatric-only sensitivity analyses closely matched the full analysis, reducing concern that inclusion of the 170 young adults materially altered the principal abnormal-result conclusions.

## 5. Conclusions

In this specialty-practice cohort, a normal or abnormal brain MRI result was documented for 37.4% of patients, and 45.7% of documented examinations carried an abnormal source-level code. Because ASD alone was not used as the reason for MRI at this practice, these results describe a clinically selected subgroup undergoing imaging for additional concerns rather than the yield of routine MRI in ASD. The routinely captured age, sex, seizure, EEG, and genetic-test variables had very limited ability to discriminate broad abnormal from normal MRI coding, whereas seizure history and abnormal EEG were associated with documented imaging utilization. Pediatric-only core and extended models closely paralleled the full-cohort analyses, suggesting that inclusion of young adults did not materially alter the principal findings. The structured subtype distribution should be interpreted only within the coded subset because coding availability differed by several measured characteristics. Overall, the study highlights the frequency and heterogeneity of MRI abnormalities encountered in patients selected for imaging in specialty care and supports future work that links richer neurological phenotyping and explicit imaging indications to clinically meaningful MRI findings. MRI decisions should remain individualized around the specific clinical question prompting structural imaging.

## Figures and Tables

**Table 1:** Cohort characteristics according to recorded MRI status.

Characteristic	Recorded MRI Result (n = 704)	No Recorded MRI Result (n = 1180)	Overall (N = 1884)	*p* Value
Age, mean (SD), years	10.97 (4.59)	10.45 (4.93)	10.64 (4.81)	0.021
Male sex, n (%)	537 (76.3)	887 (75.2)	1424 (75.6)	0.588
Seizure history, n (%)	189 (26.8)	150 (12.7)	339 (18.0)	<0.001
EEG normal, n (%)	442 (62.8)	834 (70.7)	1276 (67.7)	
EEG abnormal, n (%)	187 (26.6)	98 (8.3)	285 (15.1)	<0.001 *
EEG not tested, n (%)	75 (10.7)	248 (21.0)	323 (17.1)	
Genetic test normal, n (%)	279 (39.6)	361 (30.6)	640 (34.0)	
Genetic test abnormal, n (%)	185 (26.3)	186 (15.8)	371 (19.7)	<0.001 *
Genetic test not performed, n (%)	240 (34.1)	633 (53.6)	873 (46.3)	

* Overall Pearson chi-square test across normal, abnormal, and not tested categories. Percentages are column percentages. A recorded MRI result was defined as a normal or abnormal code; records coded not tested were classified as having no recorded MRI result. MRI, magnetic resonance imaging; EEG, electroencephalography.

**Table 3:** Logistic regression models for broad abnormal versus normal MRI coding.

Model	Predictor	Adjusted OR	95% CI	*p* Value
Core model (n = 704)	Age, per year	1.01	0.98–1.04	0.551
	Female vs. male	1.20	0.84–1.69	0.314
	Seizure history, yes vs. no	1.03	0.74–1.45	0.845
Extended complete-case model (n = 422)	Age, per year	1.01	0.96–1.05	0.762
	Female vs. male	1.37	0.87–2.16	0.174
	Seizure history, yes vs. no	0.97	0.61–1.54	0.882
	Abnormal vs. normal EEG	1.20	0.77–1.87	0.430
	Abnormal vs. normal genetic test	1.24	0.83–1.85	0.300

Normal MRI coding was the reference outcome. Confidence intervals that include 1.00 indicate statistical uncertainty and do not establish equivalence. Odds ratios concern any abnormal code and are not estimates of clinically actionable yield. OR, odds ratio; CI, confidence interval; MRI, magnetic resonance imaging; EEG, electroencephalography.

**Table 5:** Exploratory MRI categories among 159 abnormal results with structured subtype coding.

MRI Category	n	% of Coded Subset
White matter changes/gliosis	49	30.8
Cyst/CSF space	30	18.9
Ventriculomegaly/volume loss	15	9.4
Vascular/hemorrhage	12	7.5
Chiari/tonsillar ectopia	11	6.9
Cortical malformation	10	6.3
Other/nonspecific	10	6.3
Corpus callosum anomaly	8	5.0
Mesial temporal/hippocampal	5	3.1
Skull/spine/other structural	4	2.5
Pituitary/optic/sella	3	1.9
Cerebellum/posterior fossa	2	1.3

## Data Availability

The patient-level dataset is not publicly available because of patient privacy and institutional restrictions. De-identified aggregate data may be available from the corresponding author upon reasonable request and subject to institutional and legal approval.
